# Continuum of hepatitis C care cascade in prison and following release in the direct-acting antivirals era

**DOI:** 10.1186/s12954-020-00431-x

**Published:** 2020-10-20

**Authors:** Sanam Hariri, Heidar Sharafi, Mahdi Sheikh, Shahin Merat, Farnaz Hashemi, Fatemeh Azimian, Babak Tamadoni, Rashid Ramazani, Mohammad Mehdi Gouya, Behzad Abbasi, Mehrzad Tashakorian, Ramin Alasvand, Seyed Moayed Alavian, Hossein Poustchi, Reza Malekzadeh

**Affiliations:** 1 Liver and Pancreatobiliary Diseases Research Center, Digestive Disease Research Institute, Shariati Hospital, Tehran University of Medical Sciences, N. Kargar St., 14117 Tehran, Iran; 2 Middle East Liver Diseases (MELD) Center, Tehran, Iran; 3 Section of Genetics, International Agency for Research on Cancer, Lyon, France; 4 Centre for Communicable Diseases Control, Ministry of Health and Medical Education, Tehran, Iran; 5 Health and Treatment Directorate of Prisons and Security and Corrective Measures Organization, Tehran, Iran

**Keywords:** Hepatitis C virus (HCV), HCV treatment, Linkage to care, Prison healthcare, Harm reduction, HCV elimination

## Abstract

**Background:**

People with criminal justice involvement contribute remarkably to the rising hepatitis C virus (HCV) burden; however, the continuum of care is a major barrier to prison-based programs. We aimed to evaluate a comprehensive HCV care model in an Iranian provincial prison.

**Methods:**

Between 2017–2018, in the Karaj Central Prison, newly admitted male inmates received HCV antibody testing and venipuncture for RNA testing (antibody-positive only). Participants with positive RNA underwent direct-acting antiviral (DAA) therapy (Sofosbuvir/Daclatasvir). Sustained virological response was evaluated at 12 weeks post-treatment (SVR12).

**Results:**

Overall, from 3485 participants, 182 (5.2%) and 117 (3.4%) tested positive for HCV antibody and RNA, respectively. Among 116 patients who were eligible for treatment, 24% (*n* = 28) were released before treatment and 72% (*n* = 83) initiated DAA therapy, of whom 81% (*n* = 67/83) completed treatment in prison, and the rest were released. Of total released patients, 68% (*n* = 30/44) were linked to care in community, and 70% (*n* = 21/30) completed treatment, including 60% (*n* = 12/20) and 90% (*n* = 9/10) among those who were released before and during treatment, respectively. The overall HCV treatment uptake and completion were 89% (*n* = 103/116) and 85% (*n* = 88/103), respectively. From people who completed treatment, 43% (*n* = 38/88) attended for response assessment and all were cured (SVR12 = 100%).

**Conclusions:**

Integrated HCV care models are highly effective and can be significantly strengthened by post-release interventions. The close collaboration of community and prison healthcare systems is crucial to promote high levels of treatment adherence. Future studies should investigate the predictors of engagement with HCV care following release.

## Background

Following the introduction of highly effective antiviral agents, the hepatitis C virus (HCV) infection has become curable in the recent decade [1]; hence treatment of infected people has been introduced as a key strategy for disease prevention in communities [2]. Imprisonment and the increased risk of transmission after release remarkably contribute to the rising HCV burden worldwide [3, 4]. Accordingly, many countries are scaling up prison-based programs to reach the World Health Organization (WHO) target of viral hepatitis elimination as a public health issue by 2030 [5, 6]. A few studies have reported near- or micro-elimination of HCV in specific prison settings; however, evidence around the post-release engagement with care is scarce [7].

HCV case-finding among the prison population who often are underserved by community healthcare services has appeared to be cost-effective [8]. Despite the encouragement brought by recent successes, many countries have challenges expanding HCV care among people with criminal justice involvement, and access to health services often ends with patient’s release back into community [9, 10]. Retrospective studies from the USA have shown that only 10% of formerly incarcerated patients are linked to HCV care after release [11, 12]. Short prison sentences lead to high rates of treatment discontinuations, which highlights the necessity of ensuring care continuity upon release [13]. However, the transition period is accompanied by many competing priorities that often prevent patients from ongoing engagement with care [9, 11]. Such priorities include inadequate social and financial support, which often result in a return to drug-related activities and may erode all health benefits gained during incarceration [9, 14, 15].

Lack of appropriate discharge planning for HCV treatment, as well as mental disorders and substance use treatment, results in difficulty navigating through community healthcare services after release [16, 17]. Besides, poor integration between prison and community is another obstacle that can hinder immediate linkage to care and contribute to the cycle of suboptimal achievement of HCV elimination programs [18]. Community-based organizations and NGOs can play an invaluable role in the community reintegration of offenders [19]. To date, studies on linkage to care from incarceration have been mainly focused on people with HIV infection, and a variety of strategies, including case management and patient navigation, have been introduced to facilitate the transition period for these patients [15, 20]. Although developing effective care models require knowledge on the gaps in continuity of care and potential solutions [11], evidence lack around feasibility and efficiency of such interventions among HCV patients [21, 22]. Community reintegration and post-release continuity of care are current priority areas for prison healthcare research [23].

To date, no study is published on the effectiveness of HCV interventions among people with criminal justice involvement in low- or middle-income countries [24]. In recent decades, the Iranians Prisons Organization has adopted progressive harm-reduction policies; however, HCV screening and treatment are not yet provided routinely at correctional facilities. We aimed to implement a comprehensive HCV care model in a provincial prison in Iran, as a middle-income country.

### Methods

## Study population

This interventional study was conducted in the context of a national pilot on “Screening, diagnosis, and treatment of hepatitis C in Iranian prisons.” Between June 2017 and February 2018, all newly admitted male inmates in the Central Prison of Karaj, who aged above 18 years, were recruited given providing written consent. The exclusion criteria were hepatitis B infection, chronic kidney disease, cirrhosis, and HIV co-infection due to the antiretroviral drug interactions. After enrollment, the study was ongoing for about two years and patients were followed by June 2019 to complete treatment and response assessment. The review board of the Digestive Diseases Research Institute of Tehran University of Medical Sciences approved the study protocol.

### Study site

The Central Prison of Karaj is a large prison located in Karaj city, Alborz province, which is effectively a suburb of the capital city of Iran. This prison with 14 wards—inmates residing in 10 wards and four wards provide food and other services—is home to 6000 inmates at any given time and has approximately 30 new admissions daily; the majority are involved with drug-related charges (five out of 10 wards). A baseline behavioral survey was conducted in 2007, just before the introduction of methadone maintenance treatment (MMT) in this prison. According to that survey, the prevalence of drug use and injecting drug use was 93% and 42%, respectively; participants also reported having been incarcerated an average of five times before their current prison sentence [25]. The Central Prison of Karaj has a triangular clinic with one general practitioner, one psychologist, and several nurses who provide healthcare services, including HIV testing and methadone dispensing. However, there is no HCV screening or treatment program available.

### Sample collection

Before starting the project, several workshops were held by the study coordinators to educate the prison healthcare staff and ensure sampling methods. All inmates received a rapid diagnostic test (RDT) for the HCV antibody using a finger-stick blood specimen. Irrespective of the result, participants underwent venipuncture for another antibody testing by a fourth-generation enzyme-linked immunosorbent assay (ELISA). Blood samples were transferred daily to a reference laboratory outside. In case of discordant results, the plasma sample was re-evaluated by RDT in the laboratory to recognize testing errors in prison. On samples with confirmed positive antibody, quantitative HCV RNA test (The Artus HCV RG RT-PCR Kit, Qiagen) and genotyping were performed by reverse transcription-polymerase chain reaction (RT-PCR) followed by sequencing [26]. Sample collection procedures have been previously detailed elsewhere [27].

### Treatment in prison

All required education for treatment and monitoring were delivered by a liver specialist to the physician and nurses. The HCV coordinator in prison was responsible for receiving test results from the laboratory, confirming the accuracy of the patient’s contact information, and leading those with positive HCV RNA to the triangular clinic for pre-treatment counseling and biobehavioral assessment by questionnaire. Further evaluations, including complete blood count, liver enzymes, creatinine, and hepatitis B testing, were conducted in the prison laboratory before treatment initiation. AST to Platelet Ratio Index (APRI) was used for liver disease assessment, calculated as follows: [AST (U/l)/upper limit of normal (considered as 40 U/l)/platelet count (10^9^/l)] × 100. Patients received daily treatment with one tablet of a direct-acting antiviral (DAA) that was a locally-manufactured combination of 400 mg Sofosbuvir and 60 mg Daclatasvir (Sovodak®, Rojan Pharma, Tehran, Iran). The duration of therapy was 12 weeks for participants without cirrhosis (APRI < 2), and those with cirrhosis (APRI ≥ 2) were referred to a specialist health center outside the prison. The prison nurses were responsible for dispensing medication through directly observed therapy (DOT) in the clinic every morning.

### Treatment in community

If released during the study, patients were referred to the Alborz district health network (DHN), where several physicians and different healthcare providers are in charge. In Iran, DHN is identified as the setting responsible for providing health services at the township and rural level, under the supervision of state Universities of Medical Sciences. The prison HCV coordinator had to inform the network of patient’s releases and their contact details. Five tablets were provided at the patient’s disposal upon release, considering the time it takes to be linked to the network. DHN personnel were attempting to contact patients for appointment scheduling by reminder calls or reaching their residential address. Treatment was pursued by a general practitioner after receiving medical records from the prison.

### Study outcomes

The study outcomes include HCV prevalence and treatment uptake. Linkage to HCV care, defined as a documented visit in the network, was measured among people with positive HCV RNA who released before treatment initiation or completion. The other outcome was response assessment, measured by sustained virological response 12 weeks post-treatment (SVR12). SVR12 was defined as undetectable HCV RNA, performed by PCR on the venipuncture blood samples.

### Statistical analysis

Categorical variables were expressed as frequencies and percentages. The prevalence of HCV antibody and HCV RNA was calculated among all participants. Treatment uptake was measured among participants with positive HCV RNA testing, and treatment completion was evaluated among individuals who initiated treatment. Response assessment was based on intention-to-treat (ITT) among all people with positive HCV RNA who were eligible for treatment, and modified intention-to-treat (mITT) that included patients who had completed treatment.

## Results

Overall, 3485 newly admitted male inmates participated in the study, from whom 5.2% (*n* = 182) tested positive for HCV antibody. The prevalence of HCV RNA among all inmates was 3.4% (*n* = 117), indicating a viremic rate of 64% (*n* = 117/182) in this prison. The most frequent genotypes were 3a and 1a with 52% (*n* = 61) and 44% (*n* = 51) prevalence, respectively; other genotypes included 1b (3%, *n* = 4) and 3h (1%, *n* = 1).

Questionnaire data were available for half of the participants with positive HCV RNA (*n* = 60). The median age was 38 years (interquartile range (IQR) 34–44 years); the majority were heterosexual (91%), and had a drug-related sentence (73%). A history of previous incarceration was reported in 63%, and the mean (SD) incarceration time in the last year was 92 (147) days.

The majority had not finished high school (82%), were not currently employed (63%), had a minimum wage monthly income or below (65%), and all had a history of drug use (100%). During the last six months, one-quarter of patients had unstable housing (25%), and the majority had lived more than half of this time with people who inject drugs (PWID) (53%) and more than half of their friends were current drug users (67%). Compared to all patients, those who attended SVR testing appointments were older, had higher education, monthly income, and employment, and a lower proportion of them had a history of incarceration and drug-related sentences (Table 1).

**Table 1:** Characteristics of Karaj prison participants with positive HCV RNA testing

	Total	People attended SVR visit
Characteristics, n %	*n *= 60	*n *= 23
Age, median (IQR)	38 (34, 44)	39 (34, 45)
Male sex	60 (100%)	23 (100%)
Drug-related sentences	38 (73.1%)	13 (61.9%)
History of incarceration	19 (63.3%)	7 (53.8%)
Mean incarceration days^†^ (SD)	92 (147)	114 (158)
Sexual orientation		
Heterosexual	53 (91.4%)	20 (95.2%)
Homo/bisexual	5 (8.6%)	1 (4.8%)
Education		
Did not finish high school	49 (81.7%)	16 (69.6%)
Finished high school	10 (16.7%)	7 (30.4%)
Higher education	1 (1.7%)	0 (0.0%)
Employment		
Unemployed	38 (63.3%)	8 (44.4%)
Part-time	13 (21.7%)	6 (33.3%)
Full-time	9 (15.0%)	4 (22.2%)
Monthly income		
Minimum wage or below	39 (65.0%)	14 (60.9%)
Living wage	10 (16.7%)	5 (21.7%)
Above living wage	11 (18.3%)	4 (17.4%)
Place of residence		
Own house	4 (8.9%)	2 (10.5%)
Rental/Parents house	30 (66.7%)	13 (68.4%)
Homeless	11 (24.4%)	4 (21.1%)
Number of housings within 6 months		
One	43 (72.9%)	16 (72.7%)
Two or more	15 (25.4%)	6 (27.3%)
Lived with PWID^‡^ within 6 months		
Never	23 (40.4%)	10 (45.5%)
Less than half the time	4 (7.0%)	0 (0.0%)
Half the time or more	30 (52.6%)	12 (54.6%)
Number of friends with drug use		
None	10 (18.2%)	3 (14.3%)
Less than half	8 (14.6%)	4 (19.1%)
Half or more	37 (67.3%)	14 (66.7%)
Feeling of anxiety or depression	46 (79.3%)	16 (72.7%)
Sense of well-being^§^, mean (SD)	63 (19)	68 (21)

^†^In the previous year ‡people who inject drugs

^§^In a scale from zero to one hundred

### Drug use patterns

The median age at first drug use was 18 (IQR 15–22 years), and the majority had a history of use in the last six months (67%). From people who reported drug use in the previous month (42%, *n* = 24/57), 79% had used daily, most commonly Heroine and/or Methamphetamine (75%). Overall, 48% (28/59) had a history of injection; the median age at first injection was 20 (IQR 18–25 years), 25% (*n* = 7/28) had injected within the last six months and 14% (*n* = 4/28) within the previous month. From people with recent injection (past month), the majority had daily injection (75%), all Heroine (100%), and had shared needles or syringes (75%). People who attended SVR appointments were less likely to had injected within the last six months (9% vs. 25%, among those with history of injection) and shared needles or syringes (0% vs. 75%, among those with injection in the previous month), compared to all patients (Table 2).

**Table 2:** Drug use patterns and HCV care history among Karaj prison participants with positive HCV RNA

	Total	People attended SVR visit
Characteristics, n %	n = 60	n = 23
Drug use, ever	57 (100%)	22 (100%)
Age at first drug use, median (IQR)	18 (15, 22)	18 (16, 20)
Drug use within 6 months	38 (66.7%)	16 (69.6%)
Drug use in the last month	24 (42.1%)	9 (39.1%)
Daily use	19 (79.2%)	7 (77.8%)
Most commonly used drugs		
Heroine and/or Methamphetamine	18 (75.0%)	8 (88.9%)
Methadone	6 (25.0%)	1 (11.1%)
Injecting drug use, ever	28 (47.5%)	11 (50.0%)
Age at first injection	20 (18, 25)	20 (18, 27)
Injection within 6 months	7 (25.0%)	1 (9.1%)
Injection within the last month	4 (14.3%)	1 (9.1%)
Daily injection	3 (75.0%)	1 (100%)
Most commonly injected Heroine	3 (100%)	1 (100%)
Shared needle or syringe	3 (75.0%)	0 (0.0%)
Smoking daily, current	50 (87.7%)	18 (78.3%)
Alcohol use, ever	10 (18.9%)	5 (23.8%)
Opioid agonist therapy (OAT)		
Current	29 (55.8%)	10 (50.0%)
History, not current	17 (32.7%)	7 (35.0%)
Never	6 (11.5%)	3 (15.0%)
HCV knowledge^†^	4 (6.7%)	2 (8.7%)
HCV screening, ever	10 (16.7%)	4 (17.4%)
HCV treatment uptake, ever	3 (5.0%)	1 (5.3%)
Willingness to receive HCV treatment	53 (93.0%)	19 (90.5%)

^†^Answered three out of five questions correctly

### History of HCV care and knowledge

History of HCV screening (antibody testing) and treatment uptake was 17% (*n* = 10/60) and 5% (*n* = 3/60), respectively. Out of five questions around HCV knowledge, 7% (*n* = 4/60) answered three or more questions accurately. The majority had a strong willingness to receive HCV treatment (93%, *n* = 53/57) (Table 2).

### HCV treatment and linkage to care

One patient did not meet the criteria for treatment in prison due to concurrent HIV antiretroviral therapy. From 116 patients who were eligible for initiating treatment—all were candidates for a 12-week DAA therapy—24% (*n* = 28) were released and 72% (*n* = 83) initiated treatment in prison, including one individual who was released before treatment uptake and reincarcerated. Information on the treatment status of 5 other patients remains unknown.

**Fig. 1:**
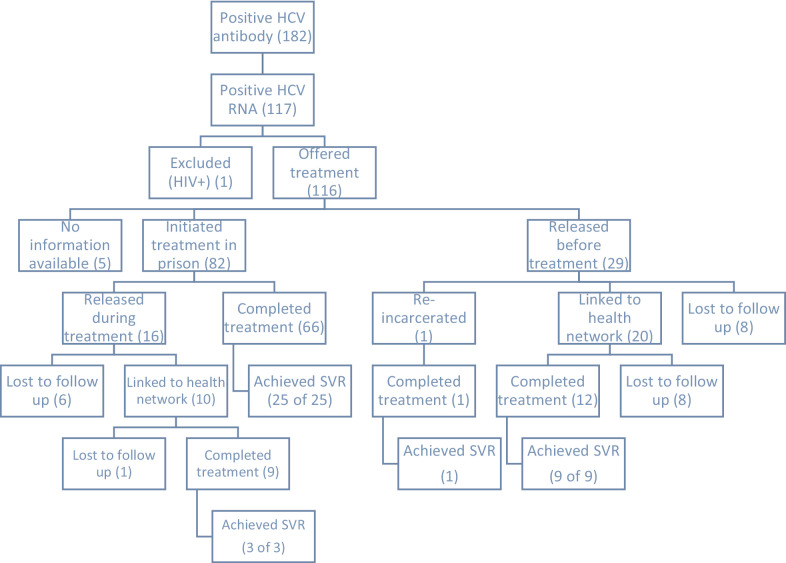
Schematic view of HCV care cascade among Karaj prison participants. SVR: Sustained virological response 12 weeks post-treatment (among those who were tested)

From patients who received treatment in prison, 81% (*n* = 67/83) completed their course on-site and the rest were released. From those who were released during treatment, 63% (*n* = 10/16) were followed by the network, and the majority completed treatment (90%, *n* = 9/10). Among patients released before treatment initiation, 71% (*n* = 20/28) were linked to HCV care in the network, and the remaining were lost to follow-up. Among those who initiated treatment in the network, 60% (*n* = 12/20) completed and the rest discontinued treatment for unspecified reasons. Therefore, among total petients who were released before or during treatment, 68% (*n* = 30/44) were successfully followed and linked to care in the community and 70% (*n* = 21/30) completed treatment (Fig. 1).

**Fig. 2:**
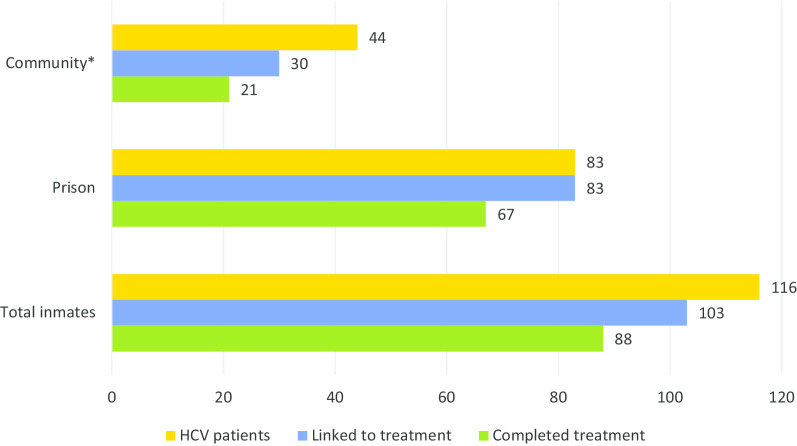
Care cascade among Karaj prison participants with positive HCV RNA, during imprisonment and after release

Overall, a total number of 103 patients initiated treatment in prison or network, resulting in a treatment uptake of 89% (*n* = 103/116). From this proportion, 85% (*n* = 88/103) completed treatment in prison or network. Forty-three percent (*n* = 38/88) of patients who had completed treatment were available for SVR assessment, who all had cured. People who initiated treatment in the community had a higher ITT SVR compared to those who initiated in prison [45% (*n* = 9/20) vs. 35% (*n* = 29/83)]. Similarly, mITT SVR for patients who completed treatment in the community was higher compared to those who completed in prison [57% (*n* = 12/21) vs. 39% (*n* = 26/67)] (Fig. 2).

## Discussion

To our knowledge, this is the first study that evaluates the impact of an HCV care program among newly admitted inmates in Iran, and one of the first studies that investigate the post-release engagement with HCV care worldwide. The prevalence of HCV antibody in this study was lower than the national estimations within prisons (5.2% vs. 8% to 28%) [28, 29], which may indicate the lower HCV infection rate among new inmates to the entire prison population. The overall engagement in treatment with 89% uptake and 85% completion rate was high, indicating the feasibility of HCV interventions among people in custody. The majority of patients who were released before or during treatment were linked to care (68%) and completed treatment (70%) in community. In comparison with retrospective studies that showed 10 to 25% linkage to HCV care after release [11, 12, 30], these findings and encouraging cure rates in our study indicate that HCV programs can be strengthened remarkably by accurate post-release patient navigation.

### HCV testing and treatment history

One-sixth of patients with available data had a history of testing, and only 5% had received treatment, indicating the missed opportunities for HCV care in correctional settings. These low rates are comparable to previous reports from Iran as well as several high-income countries [12, 31, 32]. According to a 2020 report, among people incarcerated in US prisons, only 3% have access to HCV treatment, which underlines the necessity of escalating prison-based screening and linkage to care programs [33]. Although general knowledge around HCV infection was extremely poor, willingness to initiate treatment was promising; educational initiatives during imprisonment are highly recommended and may persuade people to seek their infection status post-release.

### HCV prevalence and risk behaviors

The prevalence of HCV RNA among new inmates in this prison was slightly lower than our previous study (3.4 vs. 4.8%), which had been estimated among both new inmates and residents in Northern Iran [32]. Despite the other Iranian reports, genotype 3a was more frequent than 1a in our study population [34]. Drug-related charges were common among all patients, and the majority had high-risk friendship networks or household members. Indicators of socioeconomic marginalization and risk behaviors in the previous month were less commonly seen among people who attended SVR assessment. Combined harm reduction services, including social support and stable housing, together with expanded opioid agonist therapy (OAT) programs, are crucial to control HCV epidemic in Iran and other countries [35, 36].

### HCV treatment uptake and completion

Evidence surrounding prison-based HCV care interventions in the DAA era is scarce [37]. High treatment uptake and completion achieved in our study underlines great willingness towards treatment among people with HCV in prisons; these outcomes are comparable with another DAA-based prison study from Italy [38]. However, due to the heterogeneity of correctional settings and release patterns, effective intervention in a single prison may not be applicable in another. The median length of stay ranges from less than 48 h in jails to long-term housings in prisons, which highlights the necessity of adopting different healthcare strategies [39]. According to a US study, people who were released on parole were more likely to fill an antiretroviral therapy prescription than those with a standard release [40]. Thus, HCV programs should be tailored to the peculiar characteristics of the environment in which they are introduced [32, 39].

### HCV treatment outcomes

Previous DAA-based studies have observed high cure rates among current and former prison inmates that are consistent with our results, such as a recent report from New South Wales (NSW) (ITT 57%, mITT 92%). The lower ITT SVR in this study (42%) compared to the NSW can be explained by our two-fold higher release rates [41]. Similarly, although Pontali et al. have reported a higher ITT SVR (91%) in an Italian prison, only 6% of their patients discontinued treatment due to release. In a Scottish research, SVR assessment showed similar results for people who initiated treatment in community and prison (63% vs. 61%) [42], and a higher response was observed among people who were not released or transferred, compared to those who were released during treatment (75% vs. 45%). We observed slightly better ITT outcomes for those who commenced therapy in the community than prison (45% vs. 35%), which can be partly explained by a higher likelihood of adherence to treatment for people who are reached by the health networks after community return, compared to all released inmates. The ITT SVR among former inmates who initiated treatment in community was similar to a study from New York City jails (45% vs. 41%); however, mITT SVR in our study was higher than their observed cure rates (57% vs. 47%) [15]. This difference may suggest a lower risk of reinfection or treatment failure in the Iranian community compared to the USA. These comparisons highlight the significant impact of release patterns on treatment response assessment and its interpretations in different settings, which could incorporate into a better prison- and community-based HCV planning.

### Post-release HCV care

There is a growing body of evidence on successful transitional programs to engage patients with healthcare services after release—mainly conducted by community-based providers and NGOs—ranging from reminder calls to intensive case management [43]. Three studies from the USA have reported that only one-quarter of patients who returned to the community were linked to HCV care after incarceration [15, 30, 44]. However, we showed that more than two-thirds of patients could be linked to care following release, highlighting the critical role of active patient navigation in engaging patients with post-release care. The period of leaving incarceration is a particularly vulnerable time and many people may not receive sufficient long-term support during this period, which may lead to poor health outcomes, including treatment failure and reinfection [45]. Retention in treatment is also essential to prevent the risk of developing drug resistance [46]. Due to the similar competing priorities, factors that are considered as facilitators among people with HIV can be applied to the formerly incarcerated population with HCV to obtain synergistic effects. These include treatment for substance use and mental disorders, transportation assistance, offer drug-free transitional housing, and peer support [11, 22, 47]. Unfortunately, we were not able to provide such facilities in our study due to budget limitations.

## Limitations

The main limitation of this work was the lack of close observation on the study procedures. To provide real-world information, we aimed to assign the entire work to prison staff and community providers, which resulted in some shortcomings in patient navigation and data collection, including the loss of several medical records. Some staff changes in prison interrupted our data collection process, and tracking down all questionnaires was impossible due to peculiar restrictions of the prison environment. Consistent with the WHO report on Prisons and Health, the penitentiary healthcare system should work in close collaboration with community providers to ensure that treatment is not interrupted when people enter or leave prison and also transferred within the justice system [46]. As we only recruited newly admitted inmates, the interpretation of our results for prison residents should be with caution. Besides, women were underrepresented in this study.

## Conclusions

This work supports the feasibility of successful integrated HCV care models in custodial settings, strengthened significantly by post-release interventions. Establishing a multidisciplinary program through the collaboration of community and prison healthcare systems could promote health outcomes. A comprehensive approach should include appropriate discharge planning, increased referral resources, and patient navigation to encourage adherence to treatment among people who cycle through custody. More robust care models incorporating a variety of supportive services and risk reduction measures are needed to guarantee continuity of HCV care, and future studies should investigate the predictors of engagement with treatment and virological cure following release into the community.

## Data Availability

The datasets used and/or analyzed during the current study are available from the corresponding author on reasonable request.

## Abbreviations

APRI: AST to platelet ratio index
AST: Aspartate aminotransferase
DAA: Direct-acting antiviral
DHN: District health network
DOT: Directly observed therapy
ELISA: Enzyme-linked immunosorbent assay
HCV: Hepatitis C virus
HIV: Human immunodeficiency virus
IQR: Interquartile range
ITT: Intention-to-treat
mITT: Modified intention-to-treat
MMT: Methadone maintenance treatment
PWID: People who inject drugs
RDT: Rapid diagnostic test
RT-PCR: Reverse transcription-polymerase chain reaction
SVR: Sustained virological response
WHO: World Health Organization
